# Getting Lost in the Cell–Lysosomal Entrapment of Chemotherapeutics

**DOI:** 10.3390/cancers12123669

**Published:** 2020-12-07

**Authors:** Xingjian Zhai, Yassine El Hiani

**Affiliations:** Department of Physiology and Biophysics, Faculty of Medicine, Dalhousie University, Halifax, NS B3H 4R2, Canada; xn980835@dal.ca

**Keywords:** lysosomes, lysosomal entrapment, chemotherapeutics, chemoresistance, cancer therapy

## Abstract

**Simple Summary:**

Drug-resistant cancer cells survive under hostile bombardment of chemotherapeutic agents, causing cancer relapse and death. Shocking to many, chemo-resistant cells reprogram lysosomes, also known as the cellular “suicide bags”, to shield themselves from intrusion of chemotherapeutic agents and gain survival advantages. This review presents an evolutionary arms race through which cancer cells become adept at detecting and confining weak-base chemotherapeutic agents in lysosomes. We hope to facilitate translational pharmaceutical research by highlighting lysosomes as fruitful arenas to overcome chemotherapeutic resistance.

**Abstract:**

Despite extensive research, resistance to chemotherapy still poses a major obstacle in clinical oncology. An exciting strategy to circumvent chemoresistance involves the identification and subsequent disruption of cellular processes that are aberrantly altered in oncogenic states. Upon chemotherapeutic challenges, lysosomes are deemed to be essential mediators that enable cellular adaptation to stress conditions. Therefore, lysosomes potentially hold the key to disarming the fundamental mechanisms of chemoresistance. This review explores modes of action of classical chemotherapeutic agents, adaptive response of the lysosomes to cell stress, and presents physiological and pharmacological insights pertaining to drug compartmentalization, sequestration, and extracellular clearance through the lens of lysosomes.

## 1. History of Chemotherapy

Coined by Nobel Prize-winning German chemist and immunologist Paul Ehrlich, “chemotherapy” refers to the use of chemicals to treat diseases. In the early 1900s, Ehrlich and colleagues conducted high-throughput animal screenings of small molecules against syphilis, formulating the pioneering concept of the “magic bullets” underpinning molecular pathogenesis [1]. Ehrlich’s ingenious insights into the existence of concrete, specific cellular receptors responsible for the initiation and progression of diseases revolutionized pathology research and paved the way for further advancements in medicinal chemistry and drug discovery. Nonetheless, colossal endeavors in the search of efficacious compounds targeting molecular markers of clinical malignancies were to little avail until the two World Wars spanning the eras from 1910s to 1940s, where mustard gas was commonly used as a chemical warfare agent. Based on hematological studies of patients received in a base hospital in France in 1919, Edward Bell Krumbhaar first observed aberrations in the number of circulating leukocytes in soldiers exposed to varying levels of mustard gas [2]. Of note, patient fatality was documented alongside profound decrease of leukocyte count. In a subsequent study, Krumbhaar and Helen D. Krumbhaar attributed severe mustard gas poisoning as the causative agent for major disruptions in bone marrow and blood regeneration after exposure [3]. However, the antitumorigenic potential of nitrogen mustard did not become extensively scrutinized until the height of World War II following the infamous Bari Bombing. On December 2, 1943, a German reconnaissance aircraft discovered the Port of Bari situated off of the Adriatic Coast of Italy crowded with Allied ships. That same evening, German Junkers JU 88 bombers launched a bombing raid, sinking 27 ships, including the American Liberty Ship John Harvey, the detonation of which unleashed 2000 mustard gas bombs with each packed with roughly 60 pounds of such toxic agent. The exposure caused nearly a thousand American casualties and wounded many civilians, including medical staff with mustard burns. Autopsy reports found severe myeloid and lymphoid suppression among the diseased, sparking new interest to investigate the therapeutic potential of mustard gas in treating blood-based proliferative diseases. In 1946, two prominent pharmacologists Louis Goodman and Alfred Gilman along with colleagues in the Yale School of Medicine published the first clinical manuscript on the anti-neoplastic effects of nitrogen mustard, an analogue of sulfur mustard gas, and delivered promising result for its use in the treatment of lymphosarcoma, Hodgkin’s disease, and leukemia [4]. At the time, the predominant cancer therapies were surgical removal of tumor masses and radiation therapy. However, rampant tumor micro-metastases and the alarming patient relapses prompted the urgency for an alternative method of care. Gilman and Goodman’s groundbreaking research of nitrogen mustard and one its derivatives, mustine, opened up a new arena for cancer therapy. Indeed, nitrogen mustards became one of the first marketed chemotherapeutics and were later used to treat not just lymphomas but also lung carcinoma, dramatically improving patient remission and clinical outcomes [5]. Soon, the growing list of chemotherapeutic agents were utilized as adjuvant therapy post-surgery and radiation therapy to eradicate residual malignancies before being subsequently applied in combination therapy in the late 1960s and early 1970s with the goals of comprehensively targeting diverse aspects of cancer cell life cycle and simultaneously curbing the development of chemo-resistance. Notably, the remarkable speed at which chemotherapy has evolved was inseparable from several research powerhouses, such as the National Cancer Institute, the Memorial Sloan-Kettering Institute for Cancer research, Chester Beatty Institute, concretizing scholarly liaison connecting government and industry, university, military, and funding philanthropists [6].

## 2. Conventional Chemotherapeutic Compounds and Cell Death

### 2.1. Antimetabolites

Antimetabolites are among the earliest chemotherapeutic agents approved by the U.S. Food and Drug Administration (FDA). Although antimetabolites encompass any compounds that affect metabolic pathways, traditional antimetabolite antineoplastics often act as molecular mimicries of cellular nucleotides. Their uptake followed by activation by naturally present metabolic enzymes results in sequential disruptions of nucleic acid synthesis and metabolism, leading to cell death. One example is mercaptopurine (6-MP), a hypoxanthine (purine derivative) analog synthesized by Gertrude B. Elion in 1951 and rapidly approved by the FDA in 1953. Clinically efficacious for the treatment of childhood acute lymphoblastic leukemia, 6-MP serves as a prime medical archetype even according to today’s standard of care. Once the inactive pro-drug 6-MP enters the cell through solute carrier family transporters SLC28A2, SLC28A3, SLC29A1, and SLC29A2, it is then converted by hypoxanthine guanine phosphoribosyl transferase (HGPRT) into 6-thio-inosine monophosphate (TIMP), an inhibitor of de novo purine synthesis which, some of itself, can be further methylated by thiopurine S-methyltransferase (TPMT), to produce methyl-thioinosine monophosphate (MTIMP) [7]. MTIMP potently inhibits PRPP amidotransferase, which catalyzes the rate-determining step of de novo purine synthesis, resulting in decreased intracellular purine nucleotides [8]. Furthermore, the subsequent metabolism and biotransformation of TIMP yields thioguanine nucleotides (TGNs), the active drug metabolite of 6-MP, that can not only block DNA and RNA synthesis upon incorporation into the genome, but also render DNA-6-TG substitution constructs susceptible for cytotoxic modifications, alter the interactive dynamics of nucleotide metabolic feedback loops, and elicit further DNA damage responses [9]. Interestingly, studies have confirmed that genetic deletion of genes involved in the mismatch repair (MMR) pathways results in 6-MP resistance in leukemia models [10,11]. Although mechanistically distinct, the overall effects of many antimetabolites synthesized between 1950s and 1990s are manifested in the disruption of nucleotide production and metabolism, and imposition of S-phase genotoxicity, primarily via DNA chain termination and replicative blockage [12,13]. With the establishment of these so-called “first-generation” antimetabolites and the accruing knowledge of their clinical performance, the therapeutic design of modern antimetabolites seem to emphasize on addressing chemo-resistance, exploring novel modes of action, potentiating anti-tumor effects of existing anti-metabolites via drug combination, and limiting adverse side effects [14].

### 2.2. Alkylating Agents

Alkylating agents primarily exert their anti-neoplastic effects by destroying the genomic integrity of proliferative cells. Through either uni-molecular (SN1) or bimolecular (SN2) nucleophilic substitution reactions, alkylating agents effectively instigate covalent modifications of electron-dense reactive centers of nitrogen and oxygen atoms on DNA substrates, compromising DNA replication and giving rise to apoptotic or necrotic cell death [15]. Among the oldest class of alkylating agents are nitrogen mustards, namely *N*-methyl-bis(2-chloroethyl) amines. Such compounds first undergo an SN2 intramolecular cyclization reaction, forming the structurally strained and functionally reactive aziridinium cation. The positively charged and electrophilic nitrogen of the aziridinium cation is then subjected to nucleophilic addition by the lone pair of electrons on the seventh nitrogen (N7) of a guanine base ring, resulting in aziridinium ring opening and physically conjugating one arm of the nitrogen mustard to one strand of the DNA. Such process repeats itself for the other arm of the nitrogen mustard, effectively cross-linking DNA strand(s) and forming DNA adducts with ensuing disruptions in mitotic division. Alkylating agents whose mechanisms of action exemplify the aforementioned paradigm are consequently termed bifunctional alkylating agents, which are relatively well characterized but only make up a tiny portion of all alkylating chemotherapeutics. Because of their high reactivity, mustard agents may induce overwhelming side effects, such as acute nausea and vomiting, which patients can experience up to 20 times per day, alopecia (hair loss), oral complications, skin irritation, diarrhea, and infertility [16]. Furthermore, representing the predominant sub-category of alkylating agents, mono-functional alkylating agents (temozolomide, *N*-methyl-*N*-nitrosoguanidine, procarbazine) cause localized *N* and O-alkyl adduct formation on only one reactive center of DNA substrates, giving rise to a series of toxic and/or mutagenic modifications, such as 7-methylguanine (7meG), 3-methyladenine (3meA), O6-methylguanine (O6meG), and less frequently, O4-methylthymine (O4MeT) [17,18]. Seemingly petty, alkyl additions onto DNA substrates can further develop into double-strand breaks during cellular repair, and chromosomal translocation, which results in aberrant gene expression [19]. However, shortly after their appearance, these alkylation events can be swiftly detected and counteracted by the action of specialized methyltransferases, such as O6-methylguanine-DNA methyltransferases (MGMTs) for O6meG, base-excision repair (BER) proteins, or subjected to direct removal by the alpha-ketoglutarate-dependent hydroxylase (AlkB) family demethylases, all contributing to the persistent chemo-resistance to mono-functional alkylating agents [20]. Moreover, denoted by their “alkylating-like” mode of action, platinum-based anti-neoplastic agents, instead of adding alkyl or methyl groups directly onto the DNA substrates, induce DNA intra-strand crosslinks using the molecule’s two chemical labile sites branched off of the central platinum metal, in addition to modulating the intracellular Ca^2+^ signaling network [21]. Because platinum-based anticancer agents, such as the archetypal cisplatin, may undergo hydrolytic cleavage to become reactive species, they can be extremely cytotoxic yet known to induce a vast array of repugnant side effects, such as heart failure and kidney damage, restricting intake dosage [22,23].

### 2.3. Topoisomerase Inhibitors

During DNA replication and transcription, topoisomerases relieve torsional and flexural strain that occurs distal to the replication fork and DNA helicase. To relax the supercoiled DNA, topoisomerases reversibly cut the DNA phosphodiester backbone to create space for either a single strand of DNA to rotate or DNA duplexes to pass through the breakage point. Topo 1 generally acts to reduce the number of loops, thus torsional strain, existing on one pair of the double helix, while the homo-dimeric eukaryotic Topo 2 facilitates DNA decatenation over multiple intertwined helices. The catalytic activity of Topo II, unlike that of Topo I, is powered by ATP hydrolysis and requires the binding of Mg^2+^ [24]. Since rapidly-proliferating tumor cells require hyperactive DNA synthesis machineries, cancer cells often manipulate the expression and activity of topoisomerases to drive oncogenesis; hence the urgency to design topoisomerase inhibitors [25].

To elicit cellular apoptosis, Topo 1 inhibitors stabilize Topo 1-cleavable complexes, extending the transient lifetime of genotoxic damages. First discovered in the stem wood of the Chinese tree, Camptotheca acuminate, in 1966, the polycyclic camptothecin (CPT) recognizes the DNA-topo 1 binary complex and intercalates such complex at the site of nucleophilic cleavage via an uncompetitive paradigm, physically enlarging the distance between the 5′ hydroxyl group and the 3′ phosphorous at the cleavage by approximately 8 Å. As a result, the ternary CPT-topo 1-DNA complex prevents subsequent DNA re-ligation [26,27]. This archetypal mechanism of inducing covalent DNA adducts is also similar to how some topo II poisons, such as doxorubicin, daunorubicin, and idarubicin, function [28]. The prolonged exposure of DNA free ends is followed by a cascade of apoptosis-inducing events, some of which may include collisions with and stalling of the replication forks and RNA synthesis arrest [29]. Intriguingly, the aforementioned incidences only ensue if the CPT-stabilized Topo 1-cleavable complexes are located on the template strand within a transcribed region, suggesting that the efficiency of Topo 1 toxins may be transcriptionally dependent [30]. Many topo 1-inhibiting compounds, such as irinotecan, require hepatic activation; however, the non-specific uptake of active topo I poisons (SN-38 in the case of irinotecan) sets the clinical dosing limit to approximately 0.6 mg/m^2^ BD, with side effects potentially extending to vital organs of the body [31]. For instance, the reactivation of SN38 by beta-glucuronidases of the gastrointestinal track symbiotic bacteria induces severe diarrhea [32]. Furthermore, as topo I inhibitors (topotecan and irinotecan) lead to myelosuppression, patients have elevated risks of contracting opportunistic infections due to the reduction of white blood cells.

Since ATP hydrolysis is integral to eukaryotic Topo II catalysis, some therapeutic design of Topo II inhibitors is centered on inhibiting the enzymatic ATPase activity. Halting the catalytic ATPase activity typically involves one of two mechanisms, direct competition for ATP binding or stabilization of ATP-binding domain to prevent further ATP hydrolytic cycles. Using structure-based molecular modeling, Chène and colleagues revealed that quinoline aminopurine compound 1 (QAP1) serves as a potent ATP-competitive inhibitor for both alpha and beta isoforms of topoisomerase II, preventing Topo II-mediated DNA decatenation at low micromolar concentration [33]. As an alternative to competing for ATP binding, bisdioxopiperazine, such as ICRF-87, stabilizes the dimeric conformation of ATP-bound enzymatic orientation of Topo II by bridging subunit interface [34,35]. However, bisdioxopiperazine drug resistance can easily develop upon changes of amino acid residues in the N-terminal ATPase domain [36]. However, in contrast to stabilizing the dimeric ATPase domain, resveratrol prevents topo II ATPase domain dimerization, thereby allosterically inhibiting the enzymatic ATP hydrolytic cycles [37]. These so-called “Topo II catalytic inhibitors” have garnered increasing attention from the pharmaceutical industry in recent years after scientists discovered elevated risk of patients developing secondary malignancies after being prescribed conventional topo II poisons, mainly the anthracyclines (doxorubicin, daunorubicin, idarubicin, and mitoxantrone), as a result of the dire drug-induced genotoxic and transcriptional damages [38,39,40,41].

### 2.4. Microtubule-Targeting Agents (Vinca Alkaloids and Taxanes)

Mammalian microtubules (MTs) are composed of 13 longitudinal protofilaments laterally associated with one another around a hollow core, conferring distinct plus (+) and minus (−) polarities on beta and alpha monomers, respectively [42]. The molecular basis for such arrangement is attributed to the individual alpha and beta tubulin heterodimers, acting as GTPases undergoing catalytic cycles that allow them to build up (at + end) and disassemble the MTs (at − end) per cellular demand. This dynamic instability of MTs, as described by Marc Kirschner and Tim Mitchison in 1984, maintains cytoskeletal organization and is manifested in the form of cellular “treadmilling.” Moreover, the regulated cycles of MT assembly, catastrophe, shrinkage, and rescue are especially crucial in ensuring the faithful segregation of daughter chromatids during mitosis. Any perturbations to such process result in cell death, a theme exploited in anticancer drug design.

Isolated from Catharanthus roseus, vinca alkaloids (vincristine, vinblastine, vindesine, and vinorelbine) inhibit the polymerization of both soluble and MT-associated tubulins. Vinca alkaloids are clinically efficacious in treating a constellation of malignancies, such as breast cancer, neuroblastoma, Hodgkin’s and non-Hodgkins lymphoma, lung cancer, and choriocarcinoma. Structurally, vincristine and vinblastine are composed of an identical upper velbanamine moiety and highly similar lower vindoline constituents. To inhibit microtubule polymerization, vinblastine localizes to the interface between alpha and beta tubulin heterodimers and crosslinks the alpha-subunit of one tubulin with the beta-subunit of the other, disrupting normal microtubule architecture while inducing and stabilizing spiral-like tubulin aggregates [43]. Each of the Catharanthus derivatives, namely vinblastine, vincristine, and vindesine, exhibits varying capacity to inhibit net tubulin polymerization and mediate cytostasis [44]. Intriguingly, despite functioning as MT-targeting agents, vincristine, vinblastine, and vinorelbine can also induce extensive oxidative DNA damage [45].

Another major class of anti-neoplastic MT-targeting agents is the taxanes (docetaxel, paclitaxel, etc.), inhibiting MT depolymerization and stabilizing dysregulated MT outgrowths, thereby blocking mitosis. One interesting finding of a structure-activity relationship study illustrated that modifications at C10 position of taxoids show exceptional cytotoxicity for multidrug-resistant breast cancer MCF7 cells, without exerting major impacts on sensitive cell lines, suggesting that the C10 position of taxoids might contain moieties that serve as substrates recognized by P-glycoproteins (Pgps) during drug efflux [46]. Although paclitaxel and docetaxel share similar structures and are purported to bind at the same sites on MTs, docetaxel possesses higher binding affinity for such sites and is consistently reported as having greater efficacy in stabilizing MT assembly in vitro and in vivo [47]. Of note, docetaxel had been proven effective for paclitaxel-resistant müllerian carcinoma patients [48]. However, similar to that of paclitaxel, administration of docetaxel induces prominent side effects, such as neutropenia, leukopenia, and diarrhea [49]. Furthermore, compared with docetaxel, the novel taxoid derivative cabazitaxel, besides showing greater effect of inhibiting microtubule shortening and overall microtubule dynamicity, is also active against docetaxel-resistant tumors [50,51]. Collectively, refined chemical synthesis, innovative drug delivery, and a comprehensive understanding of not just MTs themselves but also of microtubule-associated proteins (MAPs) and their proximity interactors, may dictate the future design of MT-targeting agents.

### 2.5. Cell-Fate Determination Post-Chemotherapeutic Challenge through Lysosomes

**Figure 1:**
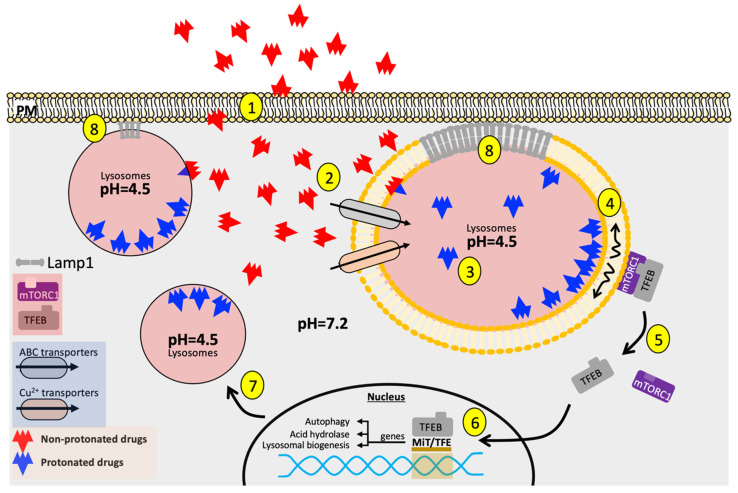
Summary: Lysosomal sequestration of weak-base chemotherapeutic compounds leads to lysosomal biogenesis and enhanced cellular clearance, further potentiating therapeutic resistance. (**1**) Lipophilic, weak-base therapeutic agents passively diffuse across the plasma membrane into the cytosol; (**2**) Unprotonated therapeutic agents enter the lysosome via passive diffusion or through ion channels (e.g., via ABC family transporters: ABCG1, ABCB2, or copper transporters: CTR1); (**3**) Therapeutic agents are rapidly protonated and become entrapped within the acidic lysosomal luminal environment; (**4**) Some protonated therapeutic compounds associate with the hydrophilic phospholipid head groups of the lysosomal membrane, disrupting lysosomal membrane fluidization; (**5**) Disruption of lysosomal membrane fluidization interferes with mTORC1 kinase activity; (**6**) Attenuated mTORC1 kinase activity facilitates nuclear translocation of transcription factor EB (TFEB); (**7**) TFEB activates Coordinated Lysosomal Expression and Regulation (CLEAR) genes, upregulating lysosomal biogenesis, autophagy, and acid hydrolase production. Significant proportion of lysosomes traffic to the plasma membrane in drug-resistant cancer cells to extrude entrapped therapeutic compounds to the extracellular matrix; (**8**) Oncogenic upregulation of glycosylated lysosome associated membrane protein-1 (LAMP1) maintains the structural integrity of lysosomal membrane and assists its docking with the plasma membrane during lysosomal exocytosis, further facilitating the efflux of therapeutic compounds.

In response to chemotherapeutic agents, tumor cells initiate a variety of pathways in the nucleus, cytosol, and plasma membrane, the overall activity of which eventually determines cell fate (Figure 1). Although lysosomes are not direct targets of these aforementioned conventional chemotherapeutic compounds, lysosomes, as the central hub for autophagy and stress signaling, decipher, integrate, and modulate cellular responses to chemotherapeutic challenges. It is well-established that autophagy presents double-edged sword effects to overall cellular survival as it can be either pro-survival or pro-apoptotic [52]. On the one hand, elevated autophagy prevents intracellular accumulation of cytotoxic metabolites while maintaining a fresh supply of organelles to carry out metabolic functions needed for tumor growth. On the other hand, excessive autophagy promotes self-degradation. To determine the nature of autophagy, as it pertains to cell survival, one needs to examine and contextualize the molecular players involved in autophagy. Elevated p53-dependent autophagy is observed in human colorectal carcinoma HCT116 cells exposed to 6-thioguanine (6-TG) and that the inhibition of autophagy results in decreased cell survival [53], highlighting the pro-survival effect of autophagy induction after genotoxic stress. This oncogenic autophagy is also observed in pancreatic ductal adenocarcinoma as suppression of autophagy through the direct interaction between ubiquitin-like protein 4A (UBL4A) and lysosome-associated membrane protein 1 (LAMP-1) inhibits pancreatic tumor migration and invasion [54]. On the contrary, increased autophagy as seen in *Beclin1*-knockin mice are protected against HER2-mediated tumorigenesis, suggesting the pro-apoptotic outcome of autophagy in HER2^+^-breast tumor pathology [55]. Furthermore, modulation of autophagy at its distinct stages in glioma cells (early-stage autophagy at the level of phosphatidylinositol 3-phosphate kinase versus late-stage autophagy during fusion between autophagosomes and lysosomes) leads to different therapeutic sensitivity to temozolomide, an alkylating agent [56]. This evidence collectively demonstrates the complexity of factors and subtlety of how dynamic progression of autophagy may underlie cellular response to chemotherapeutic challenges, thus regulating drug efficacy.

## 3. Lysosome-Mediated Acquired Chemoresistance

The acquisition of chemoresistance is a multifactorial process, integrating external growth factor/cytokine signaling, stromal-tumor interactions, internal tumor microenvironment, with the varying extent of genomic heterogeneity of the tumor mass. Cancer chemo-resistance can be broadly categorized into primary (innate) resistance and acquired resistance. Intrinsic genetic instability or genomic heterogeneity of the tumor cells, the microenvironment in which the tumor cells reside as well as the intricate host factors and patients’ comorbidities have been clinically demonstrated to confer primary resistance, rendering patients insensitive to treatment upon initial exposure to the drug [57,58]. Although this modality of resistance has been procuring increasing scientific inquiries over the recent years, primary resistance still remains a largely obscure territory. Acquired chemo-resistance, on the other hand, occurs as a consequence of the adaptive evolution of the tumor cells to sustained drug exposure. Classical facilitators of acquired chemo-resistance include apoptotic evasion, potentiated cellular efflux of chemotherapeutics, decreased drug uptake, heightened drug inactivation, alteration of drug targets and signaling outputs, increased cellular repair, and intracellular drug compartmentalization. The unifying outcome of primary and acquired resistance is the suppression of deleterious effects associated with cytotoxic outcomes after chemotherapeutic challenges. Importantly, not only do chemo-resistant cancer cells manage to metabolize cytotoxic by-products generated during chemotherapeutic challenges, they can also recalibrate redox balance and sensitivity transcriptionally to strengthen their capacity to survive in hostile environments [59,60].

Adding further complexity to this paradigm is the contentious existence of cancer stem cells driving persistent drug resistance [61]. In this section, we discuss how chemo-resistance may develop through the lens of lysosomes.

### 3.1. Organellar Physiology of Lysosomes

Lysosomes are single-membraned, dense, almost-spherical cytoplasmic vacuoles, executing compartmentalized degradation of proteins, nucleic acids, carbohydrates, lipids, and a diverse array of cellular structures through autophagy. Within the cytoplasm, lysosomes display diverse patterns of subcellular localization. Some tend to congregate in the vicinity of the nucleus and the microtubule-organizing center, forming a region known as the “perinuclear cloud,” while others are in close proximity to the plasma membrane. The subcellular localization of the lysosomes, coupled with their ability to undergo rapid intracellular trafficking upon stimulation (i.e., challenge of foreign antigens), allows them to execute a plethora of biological activities critical to maintaining cellular and organellar homeostasis. Embellished with protein signaling complexes responsive to environmental cues and regulated by transcriptional machineries, lysosomes govern the overall cellular metabolic state through dynamic regulation of anabolic and catabolic processes [54]. To create an acidic chemical environment conducive to the orchestration of phagocytic and autophagic degradation of biomolecules, lysosomes employ specialized membrane transporters, such as the vacuolar-type proton ATPases (V-ATPases) to establish proton gradient and chloride channel 7 (ClC-7), a secondary-active Cl^−^/H^+^ antiporter, to dissipate the transmembrane voltage associated with the establishment of the proton gradient by the V-ATPases [62]. The intricate coupling of ClC-7 with the lysosomal V-ATPases drives and maintains lysosomal acidification, setting the luminal pH at around 4.5–5.0, 50–60 hydrolytic enzymes known as acid hydrolases reside within the lysosomes. As their names imply, these enzymes function optimally only under acidic conditions. This selective requirement for acidity serves as a cytoprotective mechanism in case of unintended lysosomal leakage [63]. Besides V-ATPases and ClC-7, the lysosomal membrane also harbors hundreds of other membrane transporters to regulate ionic homeostasis, membrane integrity, interaction and trafficking along the cytoskeletal network, membrane fusion, ATP handling, transcriptional coupling, nutrient signaling, stress sensing, organellar contacts, and crosstalk. It is, therefore, clear that lysosomes mediate not just the proteolysis of biomolecules but also shoulder the responsibility of facilitating cellular homeostasis.

### 3.2. The Endocytic Origin and Metabolic Signaling of Lysosomes

The formation of lysosomes begins with the internalization of extracellular materials through the plasma membrane in clathrin-coated pits. As soon as endocytic cargoes gain entry to the cell in early endosomes (EEs), they are immediately in physical contact with motor proteins (i.e., dynein) connecting to the cytoskeletal networks progressing through retrograde transport. One prominent molecular attribute associated with early endosomes is the presence of Rab5 GTPase, which controls the fusion kinetics of early endosomes per se [64,65] and determines endosomal docking and maturation, when in association with other effector molecules, such as early endosome 1 (EEA1) and vacuolar protein sorting 34 (Vps34), respectively [66,67]. At this stage, moderate level of bidirectional vesicle exchange occurs between early endosomes and the acid hydrolase-containing trans-Golgi network (TGN), bestowing EEs with degradative identity as they transition and mature into late endosomes (LEs) [68,69]. As EEs mature into late endosomes, the luminal pH decreases from approximately 6.5 to 5.0 as a result of the increased relative abundance of the cytoplasmic multi-subunit V1 portion of the V-ATPases in later stages of endosome maturation [70,71]. Further endosomal maturation processes, as Huotari and Helenius elegantly summarized, encompass an increased deposition of intraluminal vesicles (ILV), a loss of capacity to recycle back to the plasma membrane, acquisition of lysosomal hydrolases, switch in fusion specificity, and association with molecular motors that lead to localization to the perinuclear cloud [68]. Eventually, late endosomes accumulate a complete set of acid hydrolases and become fully functional lysosomes, which can then, in turn, fuse with other late endosomes via the coordination of endosomal sorting complex required for transport (ESCRT), homotypic fusion and vacuole protein sorting (HOPS), and trans-soluble *n*-ethylmaleimide-sensitive factor-attachment protein receptor (SNARE) to mediate lysosomal regeneration [72].

Apart from degrading and recycling cellular contents, lysosomes also instigate intracellular signaling and organellar crosstalk. One major lysosomal nutrient signaling network is mediated through the serine-threonine kinase mechanistic target of rapamycin (mTOR), which makes up the catalytic subunit of mTOR complex 1 (mTORC1) and mTOR complex 2 (mTORC2), transducing extracellular environmental cues into intracellular responses as well as balancing autophagy with cell growth, protein synthesis, and the biogenesis of lipids and nucleotides. In mTORC1, for example, through the recruitment of the Rag GTPase obligate heterodimers (RagA/B-GTP and RagC/D-GDP) in specific nucleotide-binding states on the lysosomal membrane, amino acids indicative of nutrient availability are presented to the regulatory protein associated with mTOR (Raptor) domain of mTORC1, activating mTORC1 and allowing it to phosphorylate substrate proteins, such as the eukaryotic translation initiation factor 4E (eIF4E)- binding protein 1 (4E-BP1) and the 40S ribosomal S6 kinases (S6Ks) to stimulate the anabolic process of cap-dependent translation [73]. While promoting cellular anabolism, activated mTORC suppresses catabolic processes, central to which are lysosomal biogenesis and its various autophagic machineries. To inhibit autophagy, active mTORC1 in nutrient-rich conditions phosphorylates the Microphthalmia family of bHLH-LZ transcription factor (MiT/TFE) family member, transcription factor EB (TFEB), which, through the association with members of the YWHA (14-3-3) family of proteins, leads to cytoplasmic sequestration of this transcription factor away from the nucleus, where it transcribes a coordinated network of genes responsible for lysosomal biogenesis, lysosomal hydrolase production, and autophagy proteins [74]. However, in starvation transient receptor potential family member protein mucolipin 1 (MCOLN1) is activated and induces localized Ca^2+^ sparks near the lysosomal membrane, which is then detected by calcium/calmodulin-dependent serine-threonine phosphatase calcineurin (CaN). CaN dephosphorylates TFEB, permitting TFEB translocation into the nucleus to initiate lysosomal biogenesis in an effort to supply cellular nutrients through the degradation and recycling of biomolecules [75]. Considering the importance of mTORC-TFEB signaling axis in maintaining lysosomal homeostasis and cellular metabolism, it is unsurprising to see TFEB signaling circuitry become sabotaged during the acquisition of chemo-resistance and that the dysregulated recruitment and activity of mTORC substrates serves as the underlying etiology for various pathological conditions [76,77].

### 3.3. Lysosomal Abberations in Cancer and Chemoresistance

Organellar alterations of lysosomes have been documented in cancer. It has also been shown that members of the MiT/TFE family interact with one another to promote lysosomal biogenesis and are heavily implicated in tumorigenesis [78,79,80,81]. Considering the significance of lysosomal biogenesis and autophagic clearance in cancer, modulators of autophagy and lysosomal biogenesis, especially the MiT/TFE factors, are also regulated at the epigenetic level involving the actions of histone deacetylases (HDACs) and Myc [82]. To sustain hyperactive cell proliferation, cancer cells adaptationally enhance lysosomal activity to generate sufficient macromolecules required for cell growth and degrade damaged organelles to expedite organellar turn-over [83]. For example, in pancreatic ductal adenocarcinoma (PDA), constitutive nuclear import of MiT/TFE factors leads to upregulated lysosomal catabolism, generating autolysosome-derived pools of amino acids to sustain robust tumor growth [84]. Proteomic analysis of cisplatin-sensitive and resistant neuroblastoma cells also reveals remarkable lysosomal enrichment and proteasomal activity [85]. Autophagy inhibition, on the other hand, restores chemosensitivity to previously BRAF inhibitor-resistant brain tumor cells, highlighting the need for upregulated autophagic machineries in drug-resistant cancer cells [86]. Shedding light on the molecular basis for such upregulated lysosomal machineries comes with the study done by Zhitomirsky et al. that encapsulation of weak-base chemotherapeutic agents siramesine and sunitinib disrupts lysosomal membrane fluidization, which allows mTORC1 to dissociate from the lysosomal membrane. As mTORC1 dissociates from the lysosomal membrane into the cytosol, its kinase activity attenuates, consequentially inducing rapid TFEB translocation into the nucleus and enhancing the transcriptional output for lysosomal biogenesis to maintain acquired chemoresistance [87].

Additionally, deregulated patterns of lysosomal trafficking to the cell periphery has been observed in cancer settings, seemingly in preparation for exocytosis of entrapped weak-base chemotherapeutic compounds into the extracellular matrix (ECM) as well as subsequent metastatic dissemination through proteolytic degradation of ECM substrates [88]. Furthermore, recent evidence also indicates that copper transporters may bind to and sequester intracellular platinum-based alkylating agents, decreasing accessibility of these drugs to sites of action and causing therapeutic resistance [89,90,91]. In support of this finding, enhanced expression of copper transporter ATP7 is associated with poor clinical performance [92]. It has also been documented that unlike that found in chemo-sensitive ovarian carcinoma A2780 cells, ATP7 in cisplatin-resistant A2780 cells tends to accumulate in peripheral cytoplasmic vacuoles potentially of endosomal origin, further illustrating that alterations in subcellular trafficking of specific copper drug-binding transporters coupled with reorganization of the endo-lysosomal vesicles potentially underlie chemoresistance [93].

In addition, several lysosomal membrane proteins may also undergo oncogenic manipulations during the acquisition of chemoresistance. It is widely accepted that intra-lysosomal acid hydrolases, including phosphatases, proteases, glycosidases, peptidases, sulfatases, and lipases must be carefully protected from the cytosolic environment as massive lysosomal leakage increases cytosolic acidity and results in uncontrolled cell death [94]. To favor cell survival and circumvent cell death, cancer cells stringently upregulate lysosome associated membrane protein-1 and 2 (LAMP1 and LAMP2), both of which collectively account for approximately half of the lysosomal membrane proteins and safeguard the lysosomal membrane integrity through forming heavily glycosylated membrane barriers encapsulating the acidic luminal lysosomal environment from the cytosol [95]. Indeed, quantitative reverse transcription polymerase chain reaction of human breast cancer tissues and high-grade gliomas revealed elevated LAMP1 expression, in comparison to non-cancerous counterparts [96,97]. Intriguingly, LAMPs are not only critical to maintain structural integrity of the lysosomal membrane, they are also essential in providing a platform for lysosomal docking with the plasma membrane, facilitating lysosomal exocytosis, a theme prevalently exploited in drug-resistant cancer cells. For instance, doxorubicin-resistant sarcoma cells exhibit elevated lysosomal efflux. Sensitization of resistant sarcoma cells was achieved through LAMP1 knockdown, reinstating the function of LAMP1 in promoting lysosomal exocytosis and chemoresistance [98]. LAMP2, which, as with LAMP1, predominantly acts as a barrier against lysosomal membrane proteolysis in non-cancerous settings, can also be mobilized to the plasma membrane during early-stage tumor formation where insufficient blood vessel infiltration to the site of tumor induces hypoxic and acidic tumor microenvironment, as a cytoprotective mechanism helping tumor cells withstand chronic acidosis [99]. This finding goes hand in hand with the observation that V-ATPases that are normally embedded across the lysosomal membrane exhibit plasma-membrane localization in highly metastatic breast cancer cells to facilitate proton extrusion out to the ECM and promote ECM acidification through activation of select proteolytic enzymes [100].

Non-structural lysosomal membrane protein alterations that set the stage for chemoresistance include lysosomal acid sphingomyelinase and mTOR. Lysosomal acid sphingomyelinase (Asm) is a phosphodiesterase that mediates the hydrolytic cleavage of sphingomyeline into ceramide and free phosphocholine [101]. Ceramide acts as a secondary messenger and contributes to stress-induced apoptotic signaling, which may occur as a result of chemotherapeutic challenge. Indeed, cytotoxic response and organ damage post-cisplatin administration is attenuated in Asm-deficient (ASM^−/−^) mice in comparison to wild-type mice [102]. Also situated on the lysosomal membrane, autophagy-suppressive mTOR signaling is down-regulated in Everolimus-resistant castration-resistant prostate cancer cells, highlighting the implication of lysosomal deregulation in tumorigenesis and therapeutic resistance [103]. In addition to modifying autophagy through the canonical mTOR1/2 signaling, many tumor cells harbor rapamycin-resistant mTOR3 complex assembled by physical association with cytoplasmic ETV7 protein, exacerbating clinical responses to small-molecule drugs acting in the canonical mTOR pathway, such as rapamycin [104]. Collectively, cancer cells manipulate lysosomal membrane integrity, hijack lysosomal trafficking, and resort to the usage of non-canonical autophagic pathways while harnessing the oncogenic potential of specific lysosomal hydrolytic enzymes to support survival, invasion, and metastasis.

### 3.4. Lysosomal Entrapment of Weak-Base Compounds

Through a process known as ion-trapping, small-molecular-weight, lipophilic, and weakly basic substances are susceptible to selective retention or sequestration by acidic organelles, such as the lysosomes. The tendency for these substances to be confined within the lysosomal compartments is termed “lysosomotropism.” Mechanistically, lysosomal ion trapping primarily occurs via passive diffusion of unprotonated amine-containing drugs from the cytoplasm with relatively neutral pH environment (≈7.4) to the acidic luminal lysosome (≈4.5–5.0), autophagy, endocytosis, and lysosomal transmembrane transport system [105]. Once crossing the hydrophobic lysosomal membrane, basic amine centers undergo rapid protonation. Among the most prominent sites for lysosomal protonation are basic amine centers as, for example, contained in conventional anti-malarials, such as chloroquine and hydroxychloroquine [106]. Computer simulation has shown that the protonated portion of these aminic compounds localizes just beneath the phospholipid head groups of the lysosomal membrane and become entrapped within the luminal lysosome as the energetic barrier for charged molecules to diffuse across the lipid bilayer out to the cytosol is insurmountable under physiological settings [107]. Coincidentally, staggering numbers of aminic chemotherapeutic agents (e.g., chlorambucil, cyclophosphamide, lomustine, and doxorubicin) are susceptible to protonation and, thus, inevitably confer varying degrees of lysosomotropism due to the fact weakly basic compounds often are the predominant drug presentation following optimization of drug bioavailability and pharmacokinetic parameters. This, therefore, poses a paradoxical trade-off between cellular uptake and lysosomal partition of the drug. This, though, is not to say that the pharmacological properties of all lysosomotropic compounds will be abolished upon entry into the lysosomes. Some, such as O-methyl-serine dodecylamide hydrochloride (MSDH), in fact, possess strong detergent activity and have the potential of inducing LMP and instigating cell death [108,109]. To combat this dilemma, drug-resistant tumor cells often times manage to strengthen lysosomal membrane integrity to minimize cell death at the clinically acceptable dosage of chemotherapeutics, as discussed previously. Following entrapment of hydrophobic, weak-base chemotherapeutics, chemo-resistant cells coordinate lysosomal exocytosis, transporting antineoplastic agents out to the extracellular space and preventing intracellular deposition of cytotoxic agents [110]. Indeed, it has become increasingly clear that both the concentration of drugs and the subcellular localization of drugs at their target sites are critical determinants of therapeutic efficacy and treatment-related side effects.

Lysosomal V-type ATPase is indispensable in establishing the acidic lysosomal microenvironment, electrostatically initiating and perpetuating weak-base amine accumulation. Lysosomal acidity may be indicative of the extent of “drug retention,” suggesting a predominantly passive mechanism for drugs to enter the lysosomes. Besides promoting lysosomal acidification, V-type ATPases can also drive vesicular trafficking, thus determining the endocytic and recycling kinetics of the cell. This can have profound therapeutic implications for cancers that thrive on increased endocytic and recycling machineries. For instance, pharmacological inhibition of lysosomal V-ATPase overcomes trastuzumab (Anti-HER2 monoclonal antibody) resistance in breast cancer by impairing the elevated vesicular recycling of HER2 to the plasma membrane in trastuzumab-resistant cells [111]. Furthermore, vacuolar ATPases have also been found to promote tumor metastasis through the activation of secreted proteases [112]. These lines of evidence inarguably speak to the therapeutic potential of lysosomal ion channels in tumorigenesis and chemoresistance.

Even more intriguingly, the intracellular presence of many weak-base amines potentiates cytoplasmic vacuole formation composed of molecular assemblies of lysosomal and late-endosomal resident proteins, suggesting the existence of a built-in protein sensor for lysosomal amine accumulation [113]. Besides lysosomal vacuolation arising as an adaptive “damage-control” mechanism [114], there is also signaling network that originates from the lysosomal membrane and coordinates lysosomal biogenesis through lysosomal-nuclear crosstalk. To investigate the mechanisms associated with increased lysosomal biogenesis upon chemotherapeutic challenges, one must examine the transient receptor potential mucolipin 1 (TRPML1). Situated on the lysosomal membrane, TRPML1 is specifically activated by reactive oxygen species (ROS) produced in the adjacent mitochondria and mediates lysosomal Ca^2+^ release, which can signal through CaN to stimulate translocation of TFEB into the nucleus and result in lysosomal biogenesis [115]. Importantly, chemotherapeutic challenges often result in increased production of reactive oxygen species (ROS), which is detected by TRPML1 and used to stimulate the production of lysosomes and their associated enzymes, further supporting lysosomal entrapment of chemotherapeutics and the subsequent development of chemoresistance.

Since chemotherapeutic agents come in various shapes and forms, their chemical composition and molecular structures enable them to possess distinct lysosomal entrapment propensity. In particular, dibasic compounds exhibit higher lysosomal accumulation than monobasic compounds due to the presence of an additional basic reactive center subject to protonation, partially justifying the varying susceptibility for distinct drug molecules to undergo lysosomal sequestration [106]. Following protonation, amine-moiety-containing drug molecules will be rapidly protonated or ionized within the lysosomal compartment. Interestingly, these amines entrapped within the lysosomes demonstrate fast egress kinetics upon amine withdraw in vitro, alluding to the possibility of a secondary retro-endocytic transport machinery enabling such process [116]. Indeed, the roles of lysosomal ABC superfamily transporters have also begun to emerge in recent years [117]. For instance, most tyrosine kinase inhibitors (TKI) are substrates for ABC efflux transporters ABCB1 and ABCG2 in vitro [118]. In addition, although it is generally accepted that the P-glycoprotein, when localized to the plasma membrane, confers drug resistance by actively eliminating xenobiotics out to the extracellular space, the lysosomal localization of this protein can also contribute to organellar sequestration of exogenous substances. For instance, weak-base tyrosine kinase inhibitor sunitinib was found to localize in giant cytoplasmic vacuoles of lysosomal origin along with p-glycoproteins in several hepatocellular carcinoma cell lines [119]. Complementing this finding, using confocal microscopy and lysosomal fractionation, Yamagishi and colleagues observed a sizable reduction in lysosomal entrapment of doxorubicin and remarkable increase in nuclear localization of doxorubicin in resistant endocervical adenocarcinoma KBV1 cells upon application of P-glycoprotein inhibitor and genetic knockdown of p-glycoprotein [120]. Collectively, these studies attest to the active role of ABC family transporters in determining intralysosomal deposition of weak base chemotherapeutics.

Although lysosomal rupture due to excessive uptake of lysosomotropic agents has been reported to lead to dire cellular consequences, such as induction of intrinsic apoptosis and even necrotic cell death, lysosomes can also respond to chemotherapeutic challenges by activating appropriate compensatory measures to mitigate lysosomal stress, a central theme for manipulation by drug-resistant malignant cells [121]. For instance, Zhitomirsky and Assaraf demonstrated that lysosomal incorporation of hydrophobic weak base chemotherapeutics, such as mitoxantrone, the tyrosine kinase inhibitor sunitinib, doxorubicin, and chloroquine, triggers TFEB-mediated lysosomal biogenesis in breast cancer MCF-7 cells, instigating subsequent cancer multi-drug resistance [122]. Outside of the lysosomes, however, other organelles can also participate in stress alleviation. For instance, transcriptomic profiling of vinca-alkaloid resistant melanoma cells revealed upregulation of endoplasmic reticulum (ER) stress response genes, and that inhibition of ER stress by tauroursodeoxycholic acid (TUDCA) restores chemo-sensitivity of vinca-alkaloid-resistant cells, suggesting that ER participates in pro-survival signaling upon chemotherapeutic challenges of malignant melanoma cells [123].

### 3.5. Novel Therapeutic Strategies for Lysosome-Mediated Chemoresistance

Increasing lysosomal membrane permeability (LMP) and thus unleashing entrapped chemotherapeutic agents seems to be heavily exploited in recent years. For instance, di-2-pyridylketone 4,4-dimethyl-3-thiosemicarbazone (Dp44mT) and the clinically trialed di-2-pyridylketone 3-cyclohexyl-4-methyl-3-thiosemicarbazone (DpC) have been identified as substrates of lysosomal p-glycoproteins and subsequently induce LMP in p-glycoprotein-overexpressing cancer cells. Therefore, following doxorubicin deposition into the lysosomes, uptake of Dp44mT and DpC leads to potent apoptotic cell death through inducing ROS that eventually disintegrates lysosomal membrane in p-glycoprotein-over-expressing cancer cells, allowing doxorubicin to be re-localized to the nucleus and execute its cytotoxic effects [124]. Although this innovative modality of treatment seems promising, several reservations concerning applying Dp44mT and DpC to a wide range of chemotherapeutic agents should be considered. Apart from the limited capacity of DpC and Dp44mT to affect only the cancer cells having upregulated p-glycoprotein and the incomplete understanding of the interactome networks between chemotherapeutic agents and DpC and Dp44mT, structural mutations of the p-glycoprotein itself or increased plasma-membrane localization of p-glycoproteins may also lead to DpC and Dp44mT resistance. At the same time, it is worth noting the multifaceted nature of chemoresistance. Specifically, along with overexpression of lysosomal p-glycoproteins, drug-resistant cancer cells, as discussed previously, can also display robust structural support and maintenance of the lysosomal membrane (through overexpression of LAMP proteins) in an effort to circumvent LMP, raising a clinical question pertaining to differential drug dosing based on the heterogeneous molecular signature of each individual tumor. Various other contemporary empirical and clinical strategies to induce LMP have been documented. For instance, some have resorted to the use of a so-called “chemosensitizer,” such as chloroquine, that elevates the intra-lysosomal pH prior to doxorubicin administration [125]. In addition to serving as a lysosomal alkalinizing agent, chloroquine has also been shown to enhance endogenous nitric oxide production, which can further inhibit P-glycoprotein activity, favoring cytoplasmic localization of doxorubicin, thereby promoting its cytotoxic effects in hepatic cancer cells [126,127]. An alternative strategy to induce LMP is photodynamic therapy (PDT), where lights and a photosensitizer react in the presence of oxygen to form the cytotoxic singlet oxygen [128]. However, it has been noted that tumor cells exhibit differential tendency for apoptotic induction under PDT [129]. Furthermore, pharmacologically antagonizing heat-shock protein (HSP) 70 responsible for protecting the lysosomal membrane integrity with Pifitrin-u can also induce LMP in primary effusion lymphoma cells and provide immunogenic potential [130]. Since Pifitrin-u induces LMP and down-regulates autophagy, it is unsurprising that it also potentiates antitumor effects of chemotherapeutic agents whose efficacy is prone to attenuation by protective autophagy [131]. Although it is empirically evident that the aforementioned therapeutic paradigms achieve desirable outcomes in isolation, research on combinatorial therapy that leads to LMP induction is still lacking.

Due to the reliance of many drug-resistant cancer cells on upregulated lysosomal biogenesis to entrap chemotherapeutic agents, disrupting lysosomal homeostasis in conjunction with downstream exocytosis may be worth exploring as a therapeutic modality. One probable therapeutic intervention to target chemo-resistant cancer cells involves coupling lysosomotropic small molecule compounds with agents capable of disrupting lysosomal ionic homeostasis. This concept, nevertheless, has been empirically applied on cancer stem cells, where synthetic derivative of salinomycin with lysosomotropic potential induced intra-lysosomal iron accumulation, leading to cytotoxic ROS production and tumor cell killing [132]. Such experimental paradigm is likely based on the assumption of a uniform abundance and distribution of lysosomes and their associated components throughout the entirety of a solid tumor mass, which neither reflects nor mimics the inherent genetic heterogeneity and complex tumor microenvironment associated with a biological tumor in vivo. Therefore, outstanding clinical concerns regarding off-target effects and the acquisition of novel chemo-resistance pathways need to be addressed. Downstream of lysosomal trapping of chemotherapeutic compounds, another promising point of therapeutic interception lies during the process of lysosomal exocytosis. Genetic and pharmacological downregulation of histone deacetylase 10 (HDAC10) has been shown to decrease lysosomal exocytosis and sensitize doxorubicin-resistant neuroblastoma cells to doxorubicin-induced killing, illustrating the possibility of restoring chemosensitivity by disrupting lysosomal exocytosis [133]. Considering the varying propensity for cancers to rely on lysosomes to mediate tumorigenesis and chemoresistance, accurate diagnostic measures categorizing drug-resistant cancer subtypes by the abundance and exocytic tendency of their lysosomes is crucial.

## 4. Concluding Remarks

The revolutionary discovery of lysosomes by Christian de Duve in 1955 pioneered a field of research into biomolecular recycling, nutrient regeneration, and stress response. Since then, the world of lysosomes has garnered substantial scientific scrutiny as it represents the Achilles’ heel underpinning chemoresistance. In this review, we first discussed the advent of chemotherapy and its therapeutic trajectory, highlighting pharmacological development and mechanistic aspects for classical antineoplastic agents. Building from this foundation, we discussed the modulation of cellular responses post-chemotherapeutic challenges through the lens of lysosomes, exclusively delving into lysosome-mediated acquired chemoresistance (Table 1). Although modern therapeutic strategy centers on destabilizing the structural integrity of lysosomal membrane using single-agent interventions, combinatorial therapy involving multiple LMP-inducing agents coupled with tumor-specific lysosomal ion channel small molecule inhibitors is yet to be documented. Further research can also be directed towards elucidating the interindividual genetic and epigenetic tendency for lysosome-mediated chemoresistance, which can further optimize drug screening processes and offer predictive clinical diagnosis prior to the acquisition of chemoresistance.

## Figures and Tables

**Table 1:** Concise summary molecular modes of action of classical chemotherapeutic agents.

Drug Family	Drug Class	Drug Name	Molecular Mode of Action	References
Alkylating Agents	Nitrogen mustard	*N*-methyl-bis(2-chloroethyl) amines, Chlorambucil, Melphalan, Cyclophosphamide	Crosslinks DNA strands through initiating nucleophilic substitution with reactive centers on N and O of DNA base substrates	[15,134]
		Temozolomide, *N*-methyl-*N*-Nitrosoguanidine, Procarbazine	Forms localized *N* and O-alkyl adduct on one reactive center of DNA substrates that serves as the basis for extensive genotoxic damages, including DNA double-stranded breaks and chromosomal translocation.	[17,18,19]
	Platinum-based alkylating agent	Cisplatin	Induces DNA intra-strand crosslinks, reactive oxygen species production, cell cycle arrest, and alterations of Ca^2+^ signaling	[21]
Antimetabolites	Purine antagonist	Mercaptopurine (6-MP)	Inhibits de novo purine synthesis Produces genotoxic thioguanine nucleotides that inhibit DNA and RNA synthesis and alter their subsequent metabolism	[7,9]
	Thymidine phophorylase inhibitor	Tipiracil Hydrochloride (TAS-102)	Induces cell cycle arrest at G2 phase and DNA double-strand breaks with enhanced drug potency.	[135,136]
	Antifolate	Methotrexate, Pemetrexed	Depletes intracellular thymidine by inhibiting TYMS, DHFR, AICARFT, GART Increases intracellular dUMP and promotes its misincorporation into the DNA in inducing double-strand breaks.	[137,138]
Topoisomerase Inhibitors	Topo I inhibitor	Camptothecin	Uncompetitively cleaves DNA-topo 1 binary complex and enlarges the DNA cleavage gap, preventing further DNA re-ligation while causing collision with replication forks and eliciting apoptosis through exposure of DNA free ends.	[26,27]
		Bibenzimidazole, Terbenzimidazole	Poisons Topo I by binding to the DNA minor groove	[139]
	Topo II inhibitor	Doxorubicin, Daunorubicin, Idarubicin	Intercalates DNA and forms DNA covalent adducts Mediates Cell cycle arrest Generates reactive oxidative species	[28]
		Bisdioxopiperazine	Stabilizes ATP-bound Topo II, thereby inhibiting the enzyme’s catalytic cycles	[34,35]
		Resveratrol	Prevents dimerization of Topo II ATPase domain, thereby allosterically inhibiting the enzyme’s catalytic cycles	[37]
Microtubule-targeting Agents	Vinca alkaloid	Vincristine, Vinblastine, Vinorelbine	Inhibits microtubule polymerization Induces oxidative DNA damage	[43,45]
	Taxane	Docetaxel, Paclitaxel	Binds to and stabilizes tubulin polymers	[140]
		Cabazitaxel	Inhibits microtubule shortening and overall dynamicity	[50,51]
